# Integrated analysis of microRNA and mRNA expression profiles during the sex-differentiation sensitive period in oriental river prawn, *Macrobrachium nipponense*

**DOI:** 10.1038/s41598-017-10867-0

**Published:** 2017-09-20

**Authors:** Shubo Jin, Hongtuo Fu, Shengming Sun, Sufei Jiang, Yiwei Xiong, Yongsheng Gong, Hui Qiao, Wenyi Zhang, Yan Wu

**Affiliations:** Key Laboratory of Freshwater Fisheries and Germplasm Resources Utilization, Ministry of Agriculture, Freshwater Fisheries Research Center, Chinese Academy of Fishery Sciences, Wuxi, 214081 P. R. China

## Abstract

Male oriental river prawns (*Macrobrachium nipponense*) grow faster than females, and therefore, reach larger sizes by harvest time. Histological observations have indicated that the sex-differentiation sensitive period (which includes the formation of the androgenic gland, the testis, and the ovary) is from post-larvae (PL) developmental stage for *M. nipponense*. In this study, we prepared four microRNA (miRNA) and mRNA libraries using samples collected from sex-differentiation sensitive period (PL7 to PL16) to perform RNA-sequencing for identifying sex-related candidate miRNAs, genes, and metabolic pathways. A total of nine intersection miRNAs were identified, of which three were highly expressed in the androgenic gland, and their expression was verified by quantitative Real-Time PCR (qPCR). These three miRNAs and their 11 predicted target genes may be strong candidates for sex-related miRNAs and sex-related genes in *M. nipponense*. Five vital sex-related metabolic pathways were also identified that may regulate other sex-differentiation and sex-determination mechanisms. Finding of the study provide important insights to enhance our understanding on sex-differentiation and sex-determination mechanisms for *M*. *nipponense*.

## Introduction

The oriental river prawn, *Macrobrachium nipponense* (Crustacea; Decapoda; Palaemonidae), is a commercially important species with an annual aquaculture production of 205,010 tons^1^; it is widely distributed in freshwater and low-salinity estuarine regions of China and other Asian countries^2–6^. Like many other *Macrobrachium* species, male prawns of *M. nipponense* grow faster than females prawns^4,6^. Thus, the farming of all-male populations will be economic feasible for *M. nipponense* aquaculture. It is therefore of great importance to establish artificial sex-differentiation techniques that can be used at an early stage of gonad development for producing all male progeny in a commercial scale. A full understanding of the sex-differentiation and sex-determination mechanisms of *M. nipponense* is thus urgently needed, including knowledge of the miRNAs and genes involved at the onset of gonad differentiation.

The androgenic gland in most crustaceans produce hormones that play crucial roles in driving male sexual differentiation, the development of the testes, and the establishment of male sexual characteristics^7^. In *Macrobrachium rosenbergii*, male prawns underwent sex reversal to a female phenotype after ablation of androgenic gland. An all-male population was generated when the “reversed females” were mated with normal male *M. rosenbergii* ^7–9^. Insulin-like androgenic gland hormone (IAG) produced by the androgenic gland is an important gene for sex-determination and sex-differentiation in crustacean species, which play essential roles in male differentiation and development^10–15^. Silencing of IAG in male *M. rosenbergii* by RNA interference may also lead to complete sex reversal^16^. In *M. nipponense*, IAG is also expressed in the androgenic gland^13^.

As ablation or implantation of the androgenic gland at certain stages of development can result in sex reversal to all male or to all female^7,16–18^, studies on crustacean androgenic glands have received much attention in recent years. These studies include analysis of the expression pattern of genes expressed in androgenic gland^10–15^, and histological analysis of the androgenic gland^19–22^. In a previous histological analysis of the development of the *M. nipponense* androgenic gland, observed that the androgenic gland began to develop at PL10 and matured at PL19^23^. We hypothesized that several genes play key roles in sex-differentiation, and in androgenic gland development during these stages. Targeted analysis of miRNAs and mRNAs involved in androgenic gland developmental stages will help to identify the molecular basis of sex-differentiation and sex-determination in *M. nipponense*.

MicroRNA (miRNA) are 21–22 nucleotides (nt) long, non-coding RNAs that play an important role in the regulation of gene expression in animals and plants, by degrading target mRNA, or by repressing targeted gene translation^24^. The transcriptome is the total set of transcripts (mRNA, and non-coding RNA) transcribed during a specific developmental stage, or in response to a particular physiological condition^25^. One miRNA may regulate the expression of several genes. Alternatively, the expression of a single gene requires several miRNAs to work synchronously^24^. Several recent studies have analyzed whole transcriptomes, to establish the relationship between miRNAs and mRNAs in depth^26–28^. There is no available information on interaction networks and regulatory modes of mRNAs and miRNAs during the sex-differentiation sensitive period for any *Macrobrachium* species.

Our objectives were to identify key genes and miRNAs capable of regulating sex-differentiation across different post-larval developmental stages (from un-observation to the formation of androgenic gland) through RNA-sequencing. This study provides important insights to understand the underlying molecular mechanisms that determine sex-differentiation and sex-determination in *M. nipponense*.

## Results

### *De novo* assembly and functional annotation

**Figure 1:**
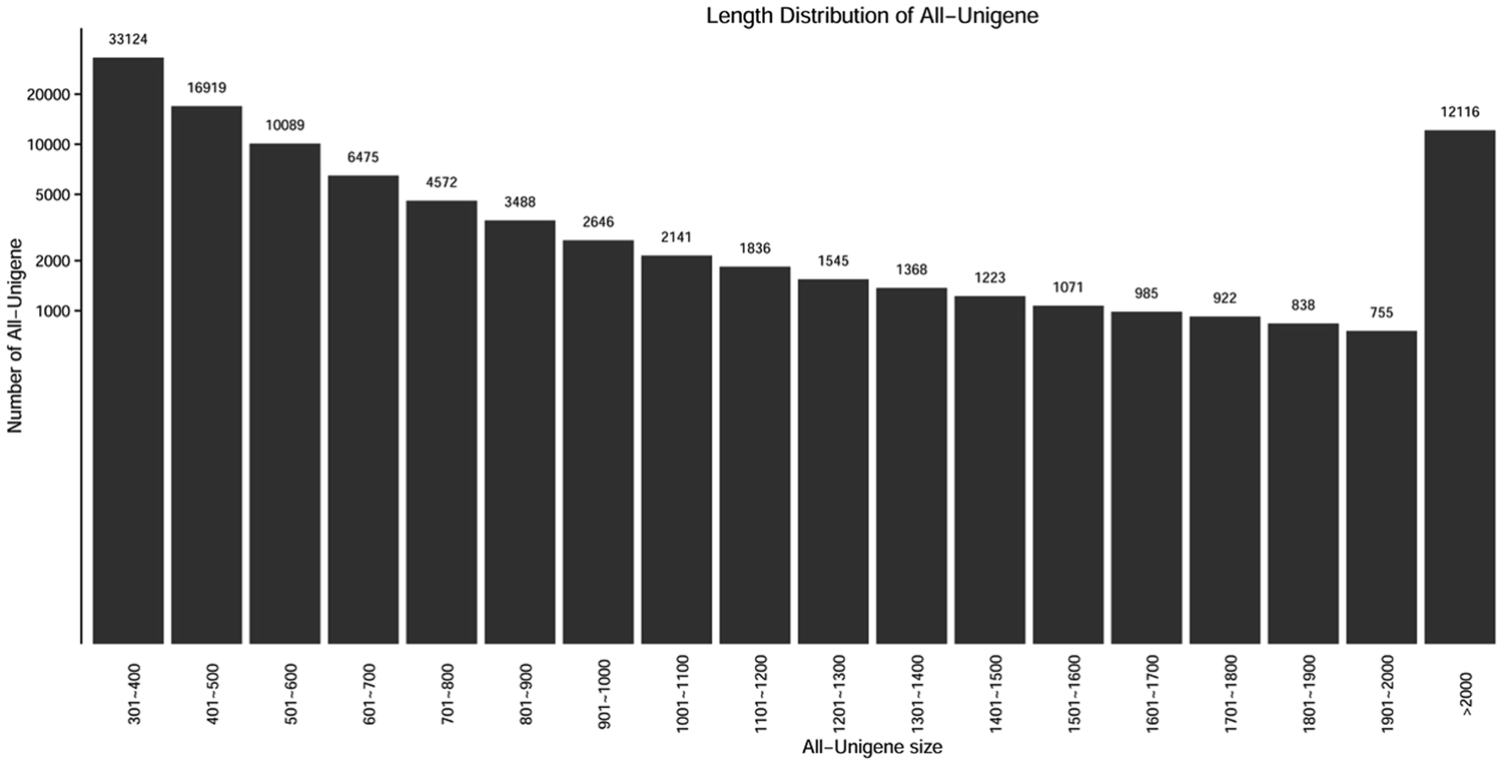
Contig length distribution of *M. nipponense* transcriptomic ESTs.

Illumina Hiseq. 2000 sequencing yielded over 56 million high-quality transcriptome reads (over 7 billion bp). *De novo* assembly of raw reads resulted in 102,113 unigenes (ranging from 301 to 32,201 bp) with an average contig length of 1,000.01 bp (Table 1). The majority of unigenes were 301–400 bp (32.43%), followed by 401–500 bp (16.57%) and >2000 bp (11.87%) in length (Fig. 1).

**Table 1:** Summary of Illumina Hiseq. 2000 sequence reads and *de novo* assembly statistics of *M. nipponense*.

	**Number**
Total number of reads for PL7	56269960
Total number of reads for PL10	56264380
Total number of reads for PL13	56296700
Total number of reads for PL16	56344980
Total number of unigenes	102113
Mean contig length	1000.01
Max contig length	32301
Min contig length	301

**Figure 2:**
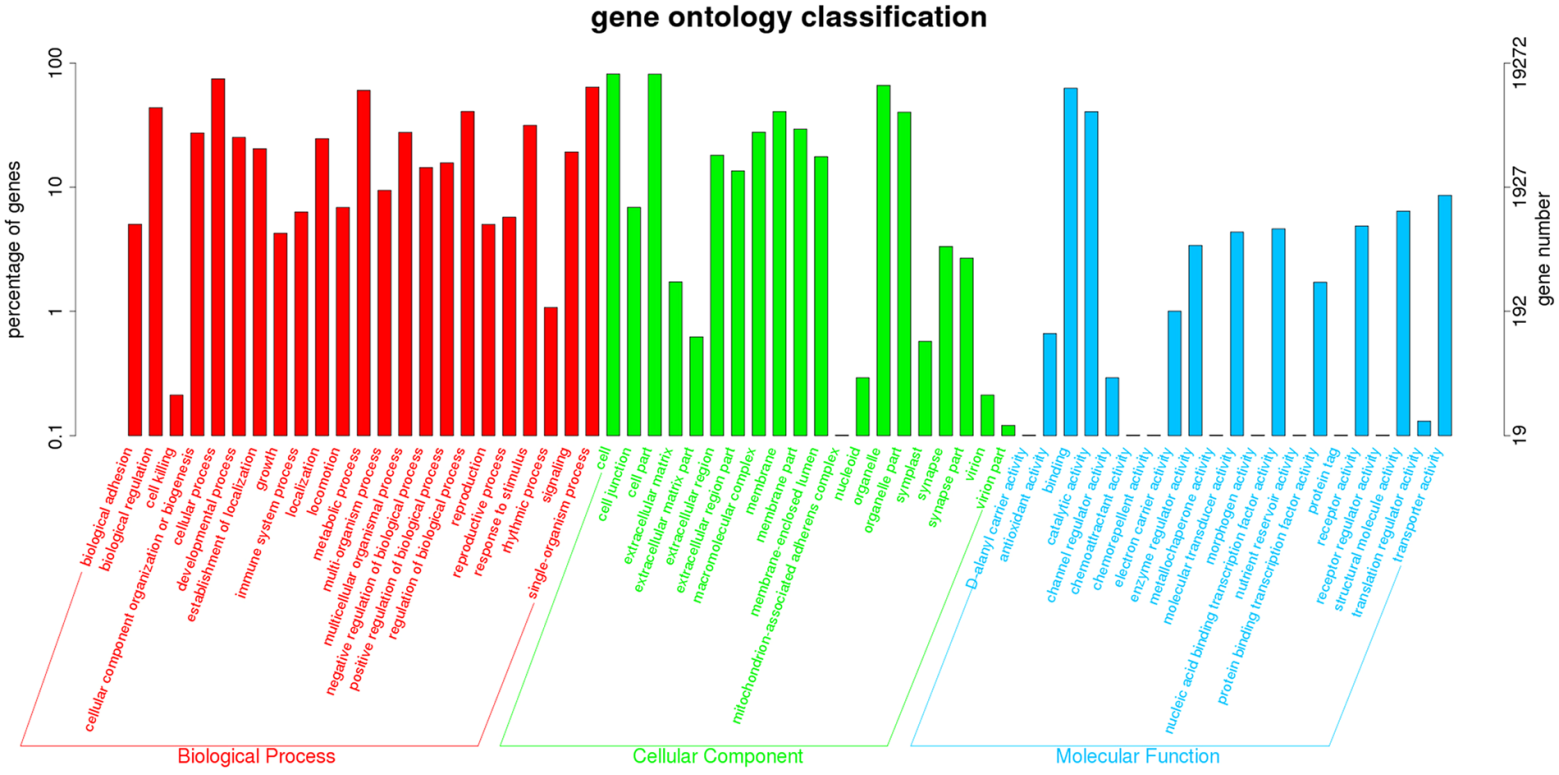
Gene ontology classification of unigenes. The left y-axis indicates the percentage of a specific category of genes existed in the main category, whereas the right y-axis indicates the number of a specific category of genes existed in main category.

To identify the putative functional roles of unigenes, they were compared with the non-redundant protein database and nucleotide sequences of the NCBI in the priority order of the Gene ontology (GO), Cluster of Orthologous Groups (COG) database and Kyoto Encyclopedia of Genes and Genomes (KEGG) database. A total of 25,026 (24.51%) unigenes were annotated in the NR database, while the other unannotated unigenes represent novel genes whose functions have not yet been identified. GO and COG analysis was performed to provide a structured and controlled vocabulary for describing gene products. A total of 19,272 (18.87%) unigenes were assigned to the GO database. Three distinct GO categories (molecular function, cellular component and biological process) were characterized with 64 functional groups (Fig. 2). A total of 18,534 (18.15%) unigenes were assigned to the COG database, and classified into 25 functional categories (Supplementary Figure 1). KEGG analysis was used to identify potential candidate transcripts in biological pathways in the ladybird. A total of 8,314 (8.14%) unigenes were assigned to the KEGG database, and were mapped onto 337 predicted metabolic pathways.

### miRNA expression profiling and screening for DEMs

**Figure 3:**
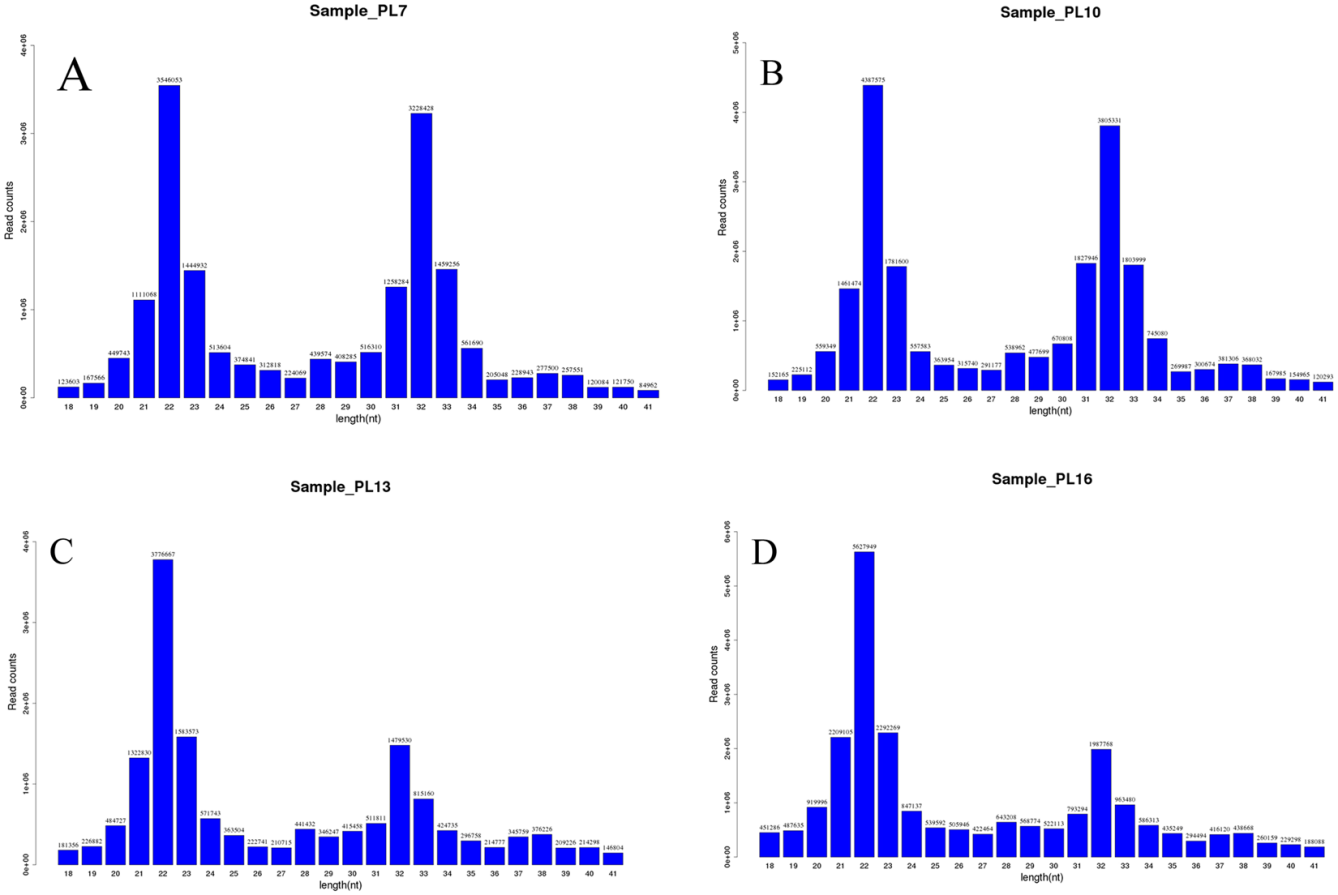
Length distribution of sequenced reads from four miRNA libraries for different developmental stages of *M. nipponense*: (**A**) PL7, (**B**) PL10, (**C**) PL13, (**D**) PL16.

In total, approximately 20 million copies of raw reads, ranging from 16–32 nt in length, were obtained for each of the four small-RNA libraries (Fig. 3). The majority of reads were 21–24 nt in length. We focused on 16–26 nt sequences for downstream analysis, because this is the typical size range of small RNAs obtained by Dicer, expect for miRNAs.

**Figure 4:**
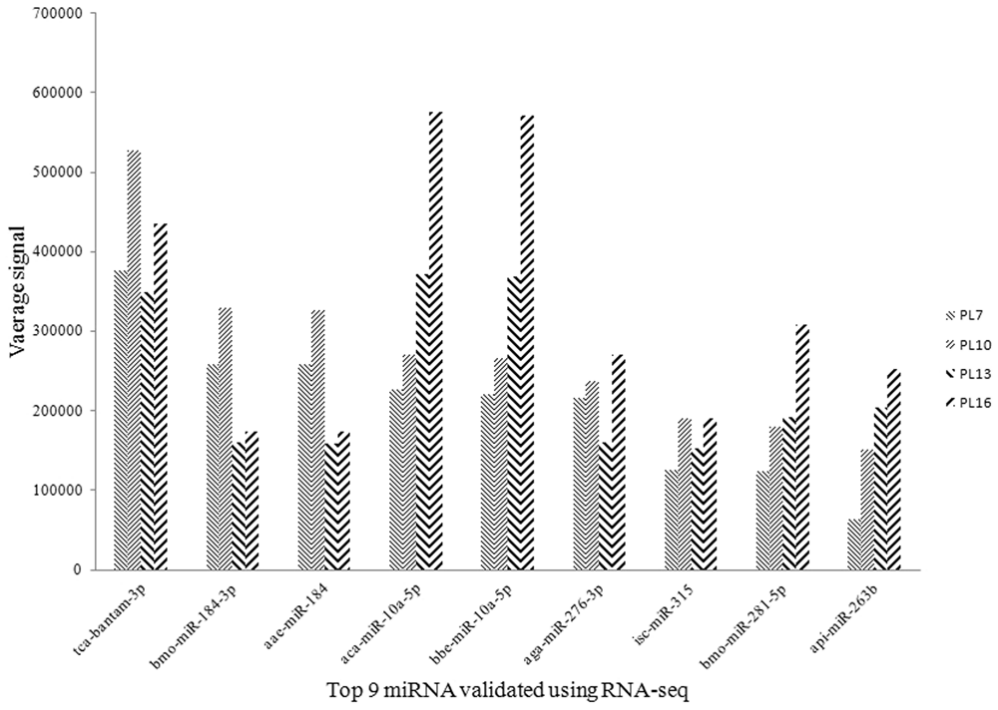
Top most abundant 9 miRNAs detected in *M. nipponense* miRNAs libraries. X-axis indicates the miRNA type. Y-axis indicates the number of conserved miRNA.

Maps that could be read were aligned to selected precursors and mature sequences in a miRBase database. The number of miRNA reads and miRNA in each small-RNA library are listed in Table 2. From these miRNAs, 1,280 miRNAs were identified in at least one of the four post-larvae developmental stages, including 108 novel miRNAs, which had not been reported before in any species. Unmapped reads were related to the Rfam database, Repbase database, mRNA database and to other RNAs (eg. rRNA, tRNA, snRNA, etc.). The top nine expressed miRNAs are shown in Fig. 4, in which the expression level of each sample group was more than 150,000 reads.

**Table 2:** Summary and analysis of *M. nipponense* miRNA sequences.

	Number of miRNA reads	Number of miRNA
PL7	3,407,789	807
PL10	4,410,771	847
PL13	4,076,881	940
PL16	6,043,628	812

**Figure 5:**
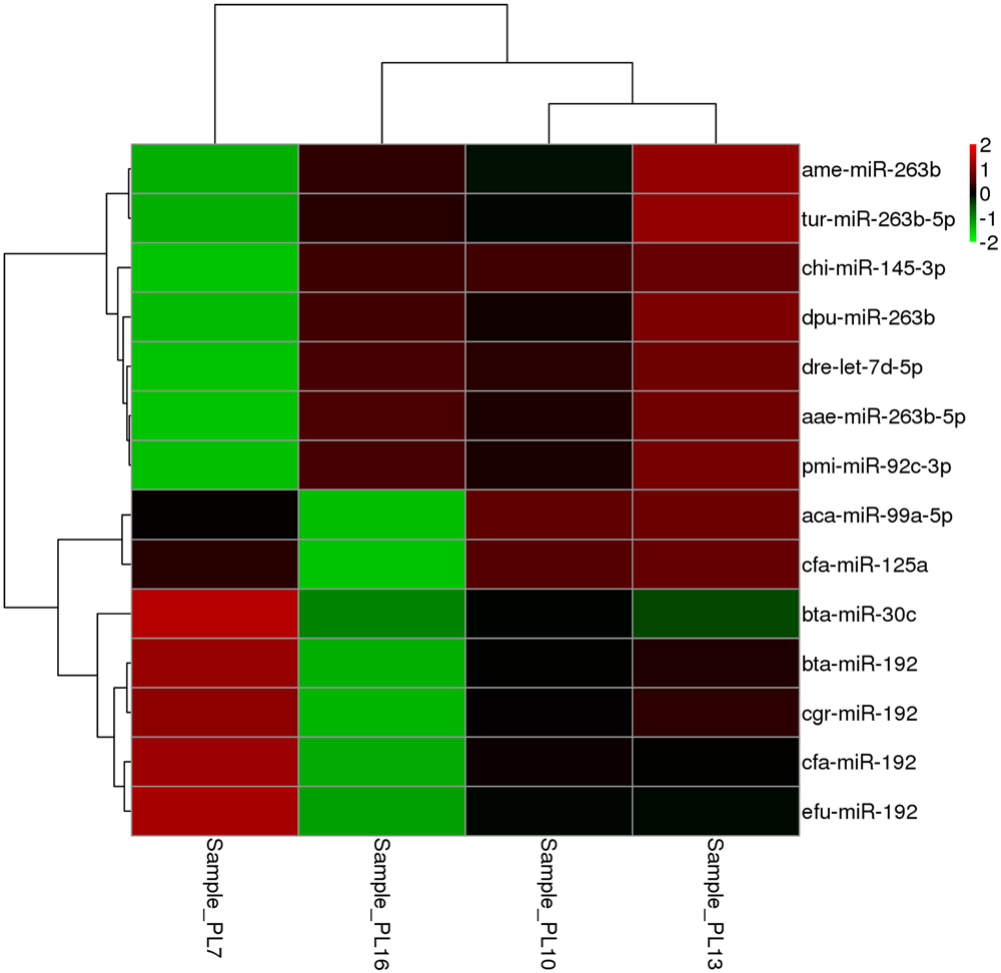
Linkage hierarchical cluster analysis of differentially expressed miRNAs in *M. nipponense* comparing the signal of the miRNAs. The color indicates the log2-fold change from high (red) to low (green), as indicated by the color scale. The names of miRNAs and the cluster to which they belong to are shown on the right side of the panel.

The miRNA expression profile of the PL7, PL10, PL13, and PL16 miRNA libraries were compared. Differentially-expressed miRNAs (DEMs) were identified based on TPM values ≥ 5 in either of the four groups, as determined by complete hierarchical linkage cluster analysis (Fig. 5). In comparison with PL10 library, 40 miRNAs were differentially-expressed in PL7 (27 up-regulated and 13 down-regulated), 101 miRNAs were differentially-expressed in PL13 (100 up-regulated and one down-regulated), and 243 miRNAs were differentially-expressed in PL16 (143 up-regulated and 100 down-regulated). Of the total DEMs, nine miRNAs were differentially-expressed in these three comparisons, and designated as “intersection miRNAs”. Intersection miRNAs included miR-30b-5p, miR-19b-3p, miR-263b, api-miR-263a, miR-125a, miR-34c-5p, miR-1175-3p, mmu-miR-470-5p and mmu-miR-871-3p, that may play important roles in post-larval development of *M. nipponense*, including the formation of the androgenic gland, the testis, and the ovaries. Furthermore, these intersection miRNAs were predicted to be candidate sex-related miRNAs.

**Figure 6:**
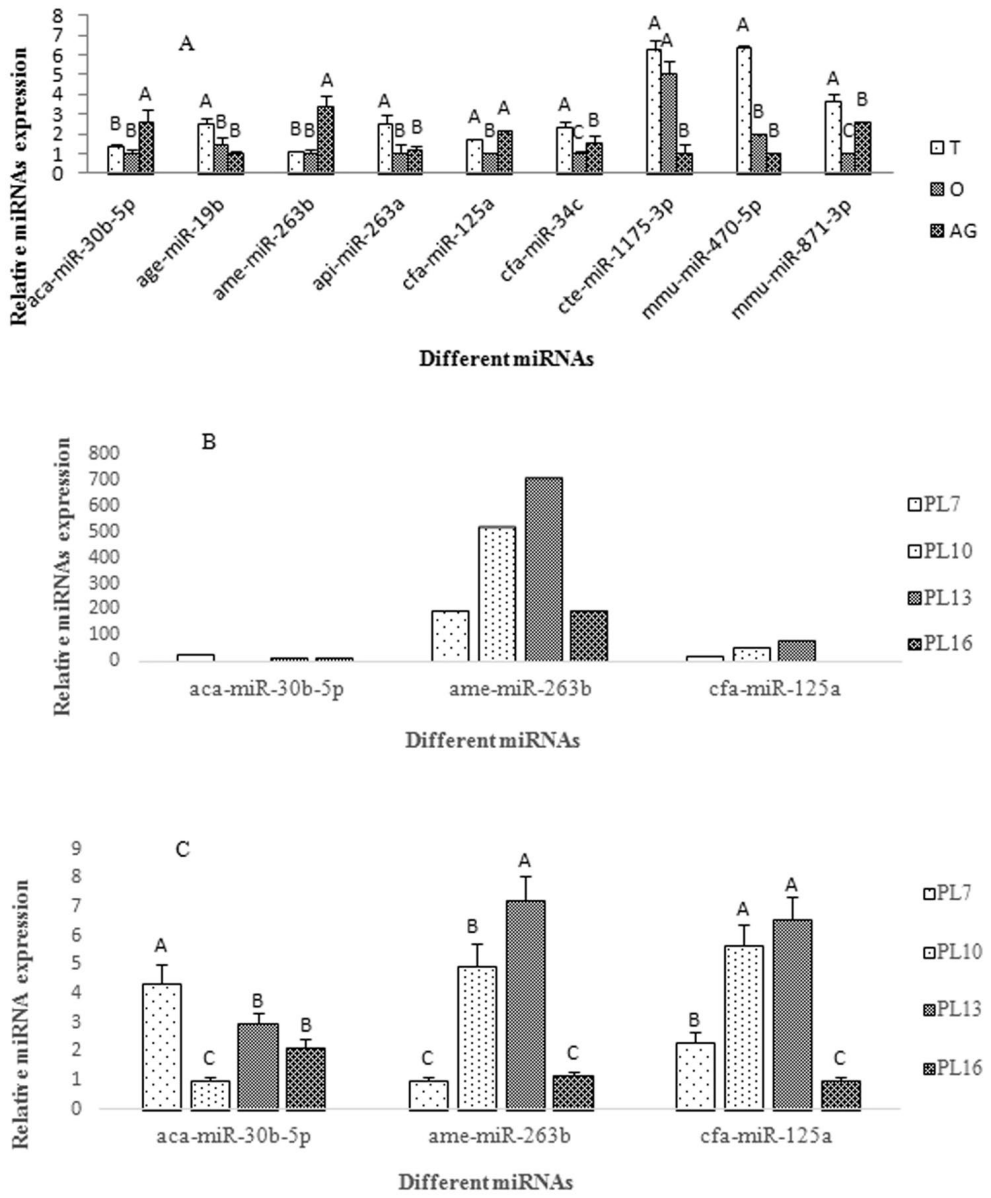
The relative expression of different intersection miRNAs revealed by real-time quantitative PCR. The amount of miRNA was normalized using U6 as control. Data are shown as mean ± SD (standard deviation) of tissues from three separate individuals. Capital letters indicate expression difference of testes from control group. A: Relative expression of 9 intersection miRNAs in testis, ovary and androgenic gland. B: Relative expression of 3 miRNAs with high expression in androgenic gland revealed by RNA-seq. C: Relative expression of 3 miRNAs with high expression in androgenic gland verified by qPCR. T: Testis; O: Ovary; AG: Androgenic gland; PL7: Post-larval developmental stages 7 and so on.

In order to further investigate the roles of the nine intersection miRNAs in sex-determination and sex-differentiation, quantitative real-time PCR (qPCR) was used to validate expression of miRNA in the testis, the ovaries, and the androgenic gland. According to qPCR analysis, three miRNAs had the highest expression level in the androgenic gland: aca-miR-30b-5p, ame-miR-263b, and cfa-miR-125a. In ovary, aca-miR-30b-5p was up-regulated by 2.59-fold, ame-miR-263b was up-regulated by 3.37-fold, and cfa-miR-125a was up-regulated by 2.12-fold (Fig. 6A). We also validated the expression of the three intersection miRNAs with the highest expression levels were observed in androgenic gland at PL7, PL10, PL13, and PL16. RT-qPCR results showed consistent patters with those of RNA-seq (Fig. 6B,C). The other six non-intersection miRNAs showed the highest expression level in the testis. No miRNA showed higher expression level in the ovaries (Fig. 6A).

### mRNA expression profiling and screening for DEGs

Comparison of mRNA expression profile among PL7, PL10, PL13, and PL16 revealed that 3,276 genes were differentially-expressed in PL7 (896 up-regulated and 2,380 down-regulated), 3,263 genes in PL13 (1.876 up-regulated and 1,387 down-regulated), and 3,021 genes in PL16 (2,182 up-regulated and 839 down-regulated) in contrast to PL10. These DEGs could be considered as important sex-related potential candidate gene for post-larval development in *M. nipponense*.

Based on the functional annotation of unigenes, a total of 33 sex-related gene families were identified in the *M. nipponense* transcriptome (Table 3). Most of the genes were identified based on comparison with published data from other species^29–37^; some genes were identified according to GO classification, to KEGG metabolic pathways, and to DEGs. Regulatory miRNAs are also listed in Table 3. The regulatory relationship needs further validation.

**Table 3:** Sex-related unigenes and their regulatory miRNAs identified in the *M. nipponense* transcriptome.

Unigene	Length	E-value	Accession number	Regulatory miRNAs
Insulin-like androgenic gland specific factor (IAG)	789-2407	0	ref|NP_563742.1|	Let-7, miR-190, miR-7, miR-71-5p, miR-9a, miR-9b
Insulin-like androgenic gland hormone binding protein	482-747	1.88E-07	gb|AJQ31852.1|	Let-7, miR-190, miR-7, miR-79, miR-9a, miR-9b
Sex-lethal	2224	4.52E-154	gb|AGI44577.1|	miR-8, miR-190, miR-315, miR-965, miR-2b, miR-311a, Let-7, miR-263b, miR-71, Mir-13a
Transformer 2	4762	2.53E-60	gb|AGI50962.1|	miR-8, miR-315, miR-279, miR-965, miR-137, miR-12, Let-7, miR-965, miR-71, miR-2a, miR-13a, miR-33, miR-87
Extra sex comb	1472	0	gb|AGI50961.1|	miR-263a-5p, miR-252a-3p, miR-315, miR-263b, miR-275, miR-263, miR-71, miR-2765-5p, miR-317
Ftz-f1	1477	1.36E-106	ref|XP_012238262.1|	miR-285, miR-9a, miR-12, miR-153, miR-307, miR-8-5p, miR-307, miR-279b, miR-190-5b, miR-10, miR-12-5p, miR-998, miR-9a, miR-12
Forkhead box protein L2 (Foxl2)	1561	1.23E-34	ref|XP_011256673.1|	miR-307, miR-10, miR-190-5p, miR-34, miR-281, miR-79, miR-263b, miR-317, miR-10, miR-305, miR-71-5p, miR-2765-5p
Sox9	3568	1.21E-37	gb|AAW51826.1|	
WNT4 protein	3315	1.56E-10	emb|CDI40100.1|	miR-92b, miR-137-3p, miR-307, miR-285-3p, miR-275,
GATA	3035	2.19E-55	ref|XP_006620188.1|	miR-9a-5p
Argonaute	3373	0	ref|XP_006620188.1|	miR-9a-5p, miR-2b, miR-87b-3p, miR-190-5p, miR-305, miR-2a, miR-993, miR-8, miR-33, miR-285-3p, miR-125, miR-iab-8, miR-263a, miR-2b, miR-315
Chormobox protein	4421	2.22E-12	gb|KFM76107.1|	
Heat shock protein 70	3078	0	gb|ACN38704.1|	miR-137-3p, miR-281-5p, miR-9a-5p, miR-281-2-5p,
Heat shock protein 90	1029	2.20E-146	gb|ADM26743.1|	miR-1000, bantam-3p
cytochrome p450	439	1.28E-21	ref|XP_782182.3|	Bantam, bantam-3p,
Cathepsin A	531	9.62E-42	gb|ADO65982.1|	miR-8-5p, miR-iab-8-5p, miR-iab-8, miR-190-5p, miR-7, miR-1a, miR-iab-4-5p, miR-7-5p, bantam-3p, miR-71, miR-9a-5p, miR-87b-3p
Cathepsin L	1039	7.56E-48	gb|AGJ03550.1|	miR-190-5p, miR-190, miR-92b, miR-281-5p, miR-87b-3p, miR-263b, miR-281-2-5p, miR-317, miR-263a-5p, miR-282-5p
Cathepsin B	1661	0	gb|AEC22812.1|	bantam-3p, miR-9a-5p, miR-2b, miR-87b-3p, miR-2a, miR-285-3p, miR-8-5p, miR-87, miR-307
Cathepsin D	1654	1.96E-67	gb|ABQ10738.1|	miR-276-5p, miR-263b, miR-8, miR-252
Cyclin A	1794	0	gb|AGG40744.1|	miR-2c, miR-2b-3p, miR-12, miR-2a,
Cyclin B	2290	0	gb|ADB44902.1|	miR-10, miR-316, miR-305, miR-10,
Cyclin D	446	5.79E-43	gb|EFX84986.1|	miR-315, miR-1, miR-190-5p, miR-8,
Cullin 3	4469	0	ref|NP_523573.1|	miR-993, miR-iab-4-5p, miR-317, miR-7-5p, miR-125, miR-7, miR-7-5p, miR-184-3p, miR-315,
ubiquitin-conjugating enzyme E2	2347	3.81E-43	ref|XP_004358782.1|	miR-1, miR-1a,
E3 ubiquitin-protein ligase	650	3.12E-16	gb|EZA55998.1|	miR-12, miR-184-3p, miR-276-5p, miR-184
ubiquitin carboxyl-terminal esterase L3	1218	2.09E-106	gb|ACO36738.1|	miR-137-3p, miR-281-5p, miR-100, miR-281-5p, miR-281-2-5p, miR-981
ubiquitin carboxyl-terminal hydrolase L3	1218	1.66E-79	ref|XP_012059656.1|	
ubiquitin carboxyl-terminal hydrolase L5	426	1.05E-45	ref|XP_004519004.1|	miR-34, miR-1a, miR-137-3p, miR-iab-4-5p, miR-137-3p, miR-iab-4-5p, miR-993, miR-34-5p
Ferritin	434	1.13E-16	gb|AGV07611.1|	miR-276a-5p,
Ferritin light-chain subunit	728	1.29E-76	gb|ACR43472.1|	miR-2a, miR-71, miR-1, miR-2b, miR-87b-3p, miR-263b, miR-190-5p, miR-305, bantam, miR-263a-5p, miR-263a
Ferritin heavy-chain subunit	1071	5.34E-73	gb|ABV60307.1|	miR-276a-5p, miR-iab-8-5p, miR-87, miR-iab-8, miR-252, miR-87b-3p
Slow tropomyosin isoform	1022	5.20E-151	miR-981	miR-184, miR-184-3p
Slow-tonic S2 tropomyosin	285	0	gb|AAS98885.1|	miR-184, miR-184-3p

### Integrated analysis of DEMs and DEGs

In this study, the target genes of each miRNA were predicted on the basis of its gene transcriptomes and Miranda algorithms. A total of 637 target genes, for the 40 DEMs between PL7 and PL10, 2,622 target genes for the 101 DEMs between PL10 and PL13, and 2,901 target genes for the 243 DEMs between PL10 and PL16 were identified. In total, 11 DEGs predicted as target genes of aca-miR-30b-5p, ame-miR-263b, and cfa-miR-125a, were identified (Table 4).

**Table 4:** The predicted targeted DEGs of aca-miR-30b-5p, ame-miR-263b and cfa-miR-125a.

miRNA	Accession number	DEG
aca-miR-30b-5p	ref|XP_011312640.1|	microtubule-actin cross-linking factor 1 isoform × 3
ame-miR-263b	ref|XP_009050915.1|	hypothetical protein LOTGIDRAFT_206222
ame-miR-263b	gb|EFX70448.1|	hypothetical protein DAPPUDRAFT_202337
cfa-miR-125a	ref|XP_011146412.1|	Dscam2
cfa-miR-125a	ref|XP_002436141.1|	phosphatidylinositol transfer protein SEC. 14
cfa-miR-125a	ref|XP_012250650.1|	coat assembly protein SEC. 16-like
cfa-miR-125a	gb|KDR22877.1|	ATP-dependent helicase brm
cfa-miR-125a	gb|EFZ11526.1|	hypothetical protein SINV_09160
cfa-miR-125a	emb|CAX48990.1|	angiotensin converting enzyme
cfa-miR-125a	ref|XP_011207921.1|	elongation of very long chain fatty acids-like protein
cfa-miR-125a	gb|AEB54796.1|	dicer 2

### Pathway analysis and GO analysis for predicted target genes

Significantly enriched GO terms identified from the differential expression analysis of PL7, PL13, and PL16, compare to PL10, consisted of 60 different functional terms. The functional terms cytoplasm, plasma membrane, and nucleus, had a higher number of transcripts than other terms.

A total of 81 differentially-expressed target genes in PL7 *vs*. PL10 were mapped to 74 different metabolic pathways. Biosynthesis of amino acids was the main metabolic pathway; three genes were mapped to this pathway. In total, 784 differentially-expressed target genes in PL10 *vs*. PL13 were mapped to 235 metabolic pathways. While 1,047 differentially-expressed target genes in PL10 *vs*. PL16 were mapped to 262 metabolic pathways. Pathways in cancer, focal adhesion, and glucagon signaling categories were the main metabolic pathways for both PL10 *vs*. PL13, and PL10 *vs*. PL16. Several genes were involved in different metabolic pathways.

## Discussion

To explore the genes, gene networks, and microRNAs that are involved in sex-differentiation and sex-determination in *M. nipponense*, we performed RNA-seq analysis during the sex-differentiation sensitive period (PL7 to PL16). To the best of our knowledge, a total of five mRNA transcriptomes^29–32,38^, and two miRNA transcriptomes^39,40^ have been reported previously for *M. nipponense*. To date, transcriptomes for post-larval developmental stages have not been reported for this species. We performed integrated analysis between miRNAs and mRNA to provide justification for selecting candidate sex-related miRNAs and sex-related genes during the sex-differentiation sensitive period. Finding of the study provide important insights into the sex-differentiation and determination mechanisms in *M. nipponense*.

### Identification of Sex-related miRNAs

In this study, 1,280 miRNAs were identified in the four miRNA libraries based on differential expression analysis, of which 108 novel miRNAs that had not been reported in any species. The qPCR analysis showed that miR-30b-5p, miR-263b, and miR-125a were highly expressed in the androgenic gland, compared to testis and ovary. These three miRNAs have been found to promote organ development in a wide range of species across the animal kingdom^41–44^. In this study, higher expression levels of miR-30b-5p, miR-263b, and miR-125a in the androgenic gland might indicate important functional roles of these three miRNAs for male differentiation and development. Thus, these three miRNAs can be considered as important drivers for sex-differentiation in *M. nipponense*. A total of 11 DEGs were predicted to be target genes of miR-30b-5p, miR-263b, and miR-125a. These DEGs may be strong candidate sex-related genes in *M. nipponense*, based on the potential functions of these miRNAs in sex-differentiation and sex-determination. However, further study is required to validate this prediction.

### Identification of sex-related metabolic pathways

*De novo* assembly revealed 102,113 unigenes in this study. Based on the functional annotation of unigenes, several sex-related metabolic pathways from other species were summarized. Ubiquitin-mediated proteolysis was the main metabolic pathway identified in this study, potentially involved with sex-differentiation and sex-determination in *M. nipponense*. The ubiquitin proteolytic system plays an important role in a broad array of basic cellular processes, including cell cycle, modulation of the immune and inflammatory responses, control of signal transduction pathways, development, and differentiation^45^. A series of DEGs involved in the ubiquitin-mediated proteolysis metabolic pathway were identified in this study, including cullin3, ubiquitin-conjugating enzyme E2 (E2), and E3 ubiquitin-protein ligase (E3) (Supplementary Figure 2). Cullin3 is a member of the Cullin gene family, which has essential effects on the cell cycle, on signal transduction, and on development. Cullin3 can directly bind to E2, and participates in various biological functions^46^. A series of E2 transcripts play essential roles in the oogenesis and spermatogenesis processes, because their expression showed significant differences at various stages of testis and ovary development^47^. Ubc9, which belongs to the E2 family, plays essential roles in embyrogenesis and oogenesis during the development of *M. nipponense* ^48^. A series of E3 transcripts are involved in the regulation of binding the target protein substrate, and in transferring ubiquitin from the E2 cysteine to a lysine residue on the target protein^49^.

Sex-lethal (*Sxl*) is the master switch gene for somatic sex-determination in *Drosophila melanogaster* that acts with the genes transformer (*Tra*), transformer-2 (*Tra-2*) and double-sex (*dbx*) to affect sex-differentiation^50^. In *M. nipponense*, *Sxl* and *Tra-2* showed similar expression patterns during embryogenesis and larval development^51,52^. A reasonable explanation could be that *Sxl* may interact with *Tra-2* to play important roles in embryogenesis, metamorphosis, somatic sexual development, and sex-differentiation in *M. nipponense*.

Foxl2 and SOX9 were identified in the *M*. *nipponense* transcriptome. Foxl2 expression is detectable in the mammalian ovary at the moment of gonadal determination^53,54^. It has been shown that Foxl2 suppressed testicular differentiation mainly through repression of the SOX9 regulatory element^55^. In addition, the absence of Foxl2 in mice leads to insufficient secretion of IGF1, resulting in developmental retardation^56^. However, recent study showed that Foxl2 may promote the male differentiation and development in *M*. *nipponense*. Tissue distributions in *M*. *nipponense* indicated that Foxl2 mRNA expression was higher in the testis and androgenic gland than that in the ovary, and higher in males than that in females in the same tissues.

Slow-tonic S2 tropomyosin and slow tropomyosin isoforms were identified in this *M*. *nipponense* transcriptome. To date, no previous studies showed that tropomyosins are sex-related genes in any other species, excepted in *M*. *nipponense* ^57^. Slow-tonic S2 tropomyosin and slow tropomyosin isoforms were reported to be highly expressed in androgenic gland of *M*. *nipponense* through comparative transcriptome analysis of that in testis and ovary^31^. Further research showed that they are novel sex-related genes in *M. nipponense*, which may play essential roles in the formation or maintenance of the structure of the androgenic gland^58^.

In conclusion, differences were demonstrated in transcriptomes and miRNAs at four different developmental stages of post-larval *M. nipponense* prawns. The integrated analysis of DEMs and DEGs suggested that three intersection miRNAs (aca-miR-30b-5p, ame-miR-263b, and cfa-miR-125a), and their predicted target DEGs, may have strong influence on sex-differentiation and sex-determination in *M. nipponens*e. In total, 5 vital sex-related metabolic pathways were identified based on the GO and KEGG analysis of DEGs, including Biosynthesis of amino acids, Pathway in cancer, Focal adhesion, Glucagon signaling categories, and Ubiquitin proteolytic system. These findings improve our current understanding of the sex-differentiation and determination mechanisms of *M. nipponense*. This study provides a basic foundation but further studies are urgently needed to investigate the actual functional roles of these selected sex-related candidate miRNAs and genes in *M. nipponense*, and how they drive sex-determination and sex-differentiation mechanism. In this study, the specimens for PL19 were not included because we do not have enough prawns after the collection of samples from PL7 to PL16 for transcriptome construction and qPCR analysis. The cultivation of specimens during post-larval development stage under lab condition leads to a dramatically low survival rate. A large number of miRNAs, mRNAs, and genes will be highly expressed at PL19 stage. Thus, many valuable information related to the completion of androgenic gland development will be lost. The candidate sex-related miRNAs and genes obtained in this study may promote the current understanding of sex-differentiation and sex-determination mechanism in *M*. *nipponense*. The comparative transcriptome analysis of PL19 with PL7-PL16 will be further performed if the prawns for PL19 are available, in order to obtain more valuable miRNAs, mRNAs, and genes related to the completion of androgenic gland development.

### Data accessibility

*M. nipponense* small-RNA libraries reads and mRNA transcriptome reads were submitted to the NCBI Sequence Read Archive, under the accession number SRR4292138 and SRR4292179, respectively.

## Materials and Methods

### Ethics Statement

We got the permission from the Tai Lake Feshery Management Council. *M. nipponense* is not an endangered species in China, thus it can be used for experimental purpose. All of the experimental programs involved in this study were approved by the committee of Freshwater Fesheries Research Center, and followed the experimental principles. Tissues from each prawn individuals were sheared under MS222 anesthesia, and efforts were made to minimize stress.

### Sample collection

Gravid females of oriental river prawn (body weights, 2.67–3.34 g) were collected from a wild population in Lake Tai, Wuxi, China (120°13′44″E, 31°28′ 22″N). All the samples were transferred to a 500 L tank and maintained in aerated freshwater at 30 °C until the embryos developed to post-larvae developmental stages. The whole individuals from different developmental stages including PL7, PL10, PL13 and PL16 were collected and immediately preserved in liquid nitrogen until used for RNA extraction. The number of specimens for each sample group was ≥ 5. The body weight of specimens was listed in Supplementary Table 1.

### RNA extraction and quality check

In total, 5 individuals for each sample group were used to extract total RNA. Total RNA was extracted for each individual separately by using UNIQ-10 Column Trizol Total RNA Isolation kit (Sangon, Shanghai, China), and then treated with RNase-free DNase I (TianGen, Beijing, China) to remove genomic DNA contamination. RNA integrity was assessed using an Agilent 2100 Bioanalyzer (Agilent Technologies, Inc.) with a minimum RNA integrity number (RIN) of 7.0.

### Library preparation and sequencing of small RNA libraries

Four equal pools (5 individuals) of total RNA were obtained from the PL7, PL10, PL13 and PL16 prawns. The samples for microRNA transcriptome analysis were prepared using a Truseq^TM^ Small RNA Sample Prep Kit (Illumina, San Diego, USA). Total RNA was ligated with proprietary 5′ and 3′ adapter. Adapter-ligated small RNA was then reverse transcribed to create cDNA constructs using Superscript reverse transcriptase (Invitrogen, CA, USA). The cDNA constructs were subsequently amplied by 15 cycles of PCR using Illumina small RNA primer set and Phusion polymerase (New England Lab, USA), and purified on 6% Novex TBE polyacrylamide gel. Sequencing cycle number is 50 and the reads length is 50 nt with single end sequencing pattern.

### cDNA library preparation and sequencing of mRNA libraries

Pools from the same prawns were used for mRNA sequencing. The samples for mRNA transcriptome analysis were prepared using a Truseq^TM^ RNA Sample Prep Kit (Illumina, San Diego, USA). mRNA was isolated from >5 μg of total RNA using oligo (dT) magnetic beads. Isolated mRNA was fragmented into smaller parts by using fragmentation buffer. Taking these short fragments as templates, first-strand cDNA was synthesized by using random hexamer-primers. RNase H, buffer, dNTP, and DNA polymerase I was used to synthesize the second-strand cDNA. Short fragments were purified using Takara’s PCR extraction Kit (Takara Bio, Inc.). Sequencing adapters were ligated to short fragments and resolved by agarose gel electrophoresis. Proper fragments were selected and purified and subsequently PCR amplified to create the final cDNA libraries. The cDNA libraries for each sample were sequenced in an Illumina Hiseq. 2000 sequencing platform. The size of each cDNA library was approximately 125 bp and both ends were sequenced.

### Bioinformatic analysis of microRNA and mRNA transcriptome data

Low quality reads (including reads shorter than 18 nucleotide) and extraneous sequences (adapter sequences) were initially filtered and removed using SeqPrep^59^ and Sickle^60^. The clean reads between 18 and 32 bp in length were used for subsequent analysis.

Non-coding RNA, such as rRNA, tRNA, snRNA, snoRNA, and scRNA were identified by aligning the obtained clean sequence to similar sequences in Rfam 11.0 and the NCBI database seraching. In order to identify known miRNA, the remnant reads were also aligned using the Blast against corresponding sequences in miRBase 21^61^ and the reference genome of *M. nipponense* unigenes of this study, allowing a maximum of two mismatches with gaps regarded as mismatches.

Miranda was used to predict the target genes of miRNA^62,63^. Target genes were predicted from blast matching against the existing EST and SRA sequences obtained from NCBI, and also by using *M*. *nipponense* mRNA transcriptome sequence obtained in this study as reference genomes. All miRNA targets were categorized into functional classes using the gene ontology hierarchy^64^.

The clean reads were obtained using NGS QC TOOLKIT v2.3.3 software^65^. Low quality reads were removed, including adaptor contamination, empty reads and low quality sequences (reads with more than 5% unknown ‘N’ or less than 25 bp). Trinity program (version: trinityrnaseq_r20131110) was used to assemble the clean reads into non-redundant transcripts^66^. The non-redundant transcripts, which are shorter than 100 bp in length and partially overlapping sequences, were removed. In order to perform the gene annotation, the resulting transcripts were then used for Blast search against the NR protein, the GO, COG, and KEGG database using an E-value cut-off of 10^−5^ ^31^. Blast2go software was used for functional annotation by GO terms^64^. Blast software was employed to perform the functional annotation against the COG^67^ and KEGG^68^ database.

### Expression analysis of miRNAs and mRNA

miRNA expression level between different miRNA transcriptome were estimated as TPM units using RSEM software^69,70^ with the following normalization formula^71^: Normalized expression = mapped read count/total reads × 10^6^. Before normalization, miRNA expression is calculated according to the number of the reads aligned to the miRBase miRNA sequence^71^. The differentially expressed genes were filtered by EB-seq algorithm. Then a FDR (False discovery rate) analysis was used under the criteria of FDR < 0.05^72^.

### Quantitative real-time PCR of miRNA

The 9 intersection miRNAs, obtained by next-generation sequencing approach, were used to perform the expression analysis in androgenic gland, testis and ovary using stem-loop qPCR. The sRNA from each sample were extracted for cDNA systhesis using a miRcute miRNA Isolation Kit (TianGen, Beijing, China) according to the manufacturer’s protocol. The specific primers with a stem-loop structure were designed following Chen *et al*.^73^. Total RNA was reverse transcribed with miRNA specific stem-loop primers using TaqMan MicroRNA Reverse Transcription Kit (Life Technologies) according to the manufacturer’s protocols. The PRISM® 7900HT Real-Time PCR System (ABI) and the Platinum SYBR Green qPCR SuperMix-UDG (Invitrogendn, 11733-038) were used to conduct real-time quantitative PCR assays. U6 was chosen as an internal control to correct for analytical variations. The primer list has been presented in Table 5.

**Table 5:** Primers used in this study.

miRNAs	Primer sequence
U6	CAAGGATGACACGCAAATTCG
aca-miR-30b-5p	CTTGGCACTGGGAGAATTCACAG
ame-miR-263b	CTTGGCACTGGAAGAATTCAC
api-miR-263a	ATGGCACTGAAAGAATTCACGGG
cfa-miR-125a	CTGAGACCCTTTAACCTGTAA
cfa-miR-34c	AGGCAGTGTAGTTAGCTGATTGC
cte-miR-1175-3p	TGAGATTCAACTCCTCCAACTGC
mmu-miR-470-5p	TTCTTGGACTGGCACTGGTGAGT
mmu-miR-871-3p	TGACTGGCACCATTCTGGATAAT
age-miR-19b	TGTGCAAATCCATGCAAAACTGA

The relative expression (fold changes) of selected miRNAs were calculated using the 2^−*ΔΔ*CT^ method^74^, and the level of significance was analyzed by one-way analysis of variance (ANOVA) with SPSS software version 18.0. Statistically significant differences were examined by paired t-test. A value of P < 0.05 was considered to be statistically significant.

## Electronic supplementary material

Supplementary information
