# Supplementary material for: Integrated analysis of microRNA and mRNA expression profiles during the sex-differentiation sensitive period in oriental river prawn, Macrobrachium nipponense
Source: Sci Rep. 2017 Sep 20;7:12011. doi: 10.1038/s41598-017-10867-0 (PMC5607309; doi:10.1038/s41598-017-10867-0)
Supplement: Supplementary file 1 — Supplementary information [file 41598_2017_10867_MOESM1_ESM.pdf]

Integrated analysis of microRNA and mRNA expression profiles during the sex-differentiation sensitive period in oriental river prawn, *Macrobrachium nipponense*

Shubo Jin[1], Hongtuo Fu[1]*, Shengming Sun[1], Sufei Jiang[1], Yiwei Xiong[1], Yongsheng Gong[1], Hui Qiao[1], Wenyi Zhang[1], Yan Wu[1]

1. Key Laboratory of Freshwater Fisheries and Germplasm Resources Utilization, Ministry of Agriculture, Freshwater Fisheries Research Center, Chinese Academy of Fishery Sciences，Wuxi 214081, P. R. China

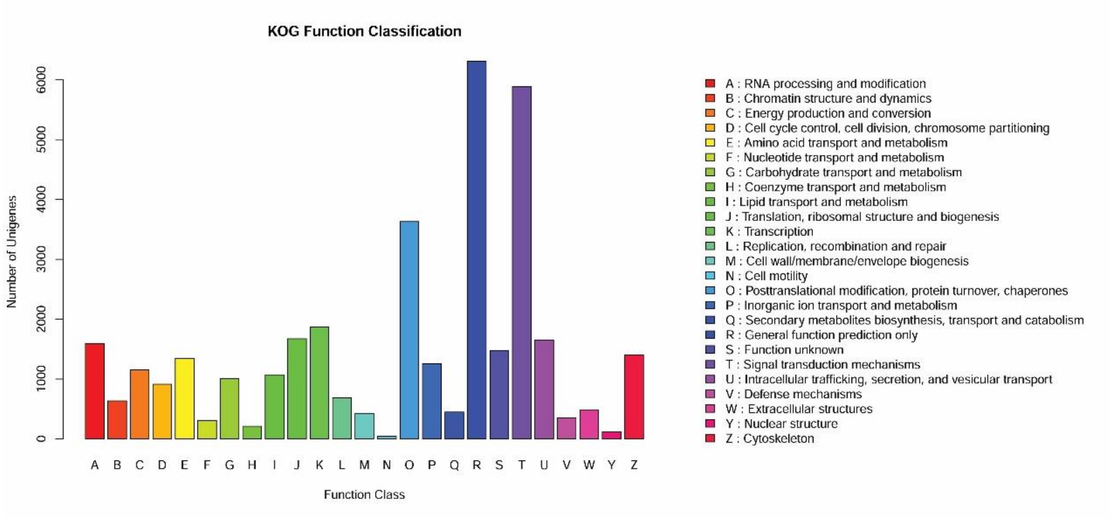

Supplementary Figure 1. Cluster of orthologous groups (COG) classification of putative proteins.

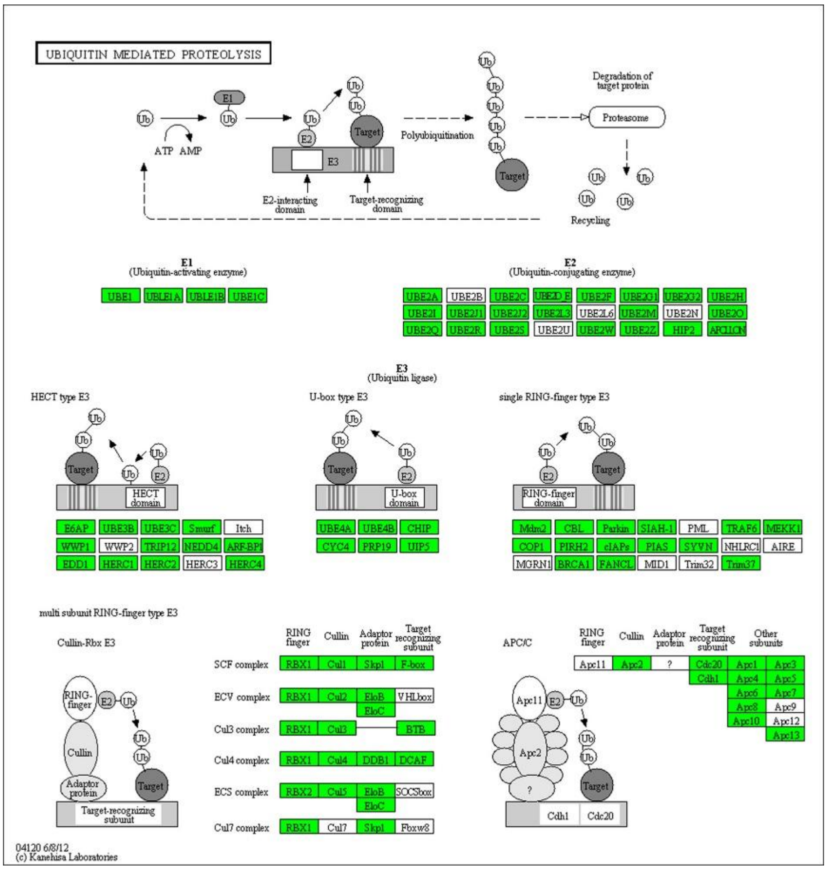

Supplementary Figure 2: Ubiquitin-mediated proteolysis metabolic pathway.

Kanehisa, M., Furumichi, M., Tanabe, M., Sato, Y., Morishima, K. KEGG: new perspectives on genomes, pathways, diseases and drugs. *Nucleic Acids Res*. **45**, D353-D361 (2017).

Kanehisa, M., Sato, Y., Kawashima, M., Furumichi, M., Tanabe, M. KEGG as a reference resource for gene and protein annotation. *Nucleic Acids Res*. **44**, D457-D462 (2016).

Kanehisa, M., Goto, S. KEGG: Kyoto Encyclopedia of Genes and Genomes. *Nucleic Acids Res*. **28(1)**, 27-30 (2000).

Supplementary Table1. The body weight of used specimens

Developmental days	Body weight (g)	Average body weight (g)
PL7	0.0032-0.0212	0.00756
PL10	0.0046-0.0190	0.0105
PL13	0.0047-0.0191	0.0123
PL16	0.0141-0.0517	0.0351
